# Impact of Using Processed Urinary Bladder Submucosa and Hydrogel Fabricated from Tendon on Skin Healing Process in Rabbits

**DOI:** 10.1155/2024/6641975

**Published:** 2024-02-06

**Authors:** Majid A. Alkhilani, Omar Tariq Hammoodi, Hasanain Abduljabbar Emran, Wissam Abdullah Alhayani

**Affiliations:** ^1^Department of Surgery and Obstetrics, College of Veterinary Medicine, University of Fallujah, Anbar, Iraq; ^2^Department of Surgery and Obstetrics, College of Veterinary Medicine, University of Baghdad, Baghdad, Iraq

## Abstract

This study was intended to evaluate the healing of skin injury by using decellularized urinary bladder submucosa scaffolds and tendon-derived hydrogel. Thirty-six adult local breeds of both sex rabbits, with an average weight of 2.0–2.5 kg, were divided randomly into three groups (12 animals for each group). All groups were subjected to an induced injury (2 cm) in diameter at the right side of the abdominal skin, the rabbits of the 1st group (A) were sutured without the application of any substance as a control group, the rabbits of the 2nd group (B) were sutured and treated with the application of decellularized urinary bladder submucosa scaffolds, and in the 3rd group (C), they were sutured and treated with the application of tendon-derived hydrogel. Postoperative care following had been done for all groups throughout the study period. Specimens from the injured skin were taken for the histopathological study on the postinjury day, 8th, 14th, 21st, and 24th. The study showed a clear effect of materials used in the treatment of wounds through a clear progression in the healing stages with a noticed superiority of the submucosa scaffold group.

## 1. Introduction

Wound is a full or partial interruption in the integrity of the skin, caused by physical injuries such as surgery, friction, or by chemicals. Also, it can occur due to diseases such as carcinoma. Sometimes, these wounds need long time for healing and repair, and for that reason, many of researchers used a lot of different materials and methods for decreasing the time of wound healing [1].

The process of wound healing is composed of incorporated cellular and biochemical cascades leading to the restoration of the functional and structural integrity of the damaged tissue [2]. The healing of the injured tissues consists of a sequence of events that occurs in overlapping stages, inflammation stage, proliferation stage, and the remodeling stage [3]. The inflammatory stage starts immediately with the hemorrhage of the wound and at first starts with vasoconstriction of the blood vessels at the site of injury which leads to homeostasis and releases a variety of mediators. The proliferative stage in which the production of collagen and granulation tissue is formed by fibroblast and angiogenesis process increased. The remodeling phase has the improvement in the components of the collagen fiber, has regeneration of the epithelial cells, and increases the tensile strength of the collagen [4]. Most of the recent studies are focusing on speeding up soft tissue and wound healing, restore the structural and morphological appearance of the skin, and avoid scar tissue formation [5]. Various therapeutic materials from different sources such as natural and synthetic compounds that are effective on the healing of the tissue, nonimmune response, and noncorrosive to the body, and with easy and low-cost preparation are used for enhancement of the wound healing process as topical and systemic drugs [6, 7]. Moreover, there are numerous biological products derived from animal sources such as amniotic membrane, equine pericardium (EP), small intestine submucosa (SIS), urinary bladder matrix (UBM), and tendon-derived hydrogel that have been used in the treatment of skin injury to work as scaffold and graft for migration and proliferation of the cell to accelerate the wound healing process [8–11]. Biocompatibility, biochemical and biomechanical properties, close contact with the cells, and nonimmune response are the ideal characteristics of the graft material used in the accelerating of wound healing [12]. Hydrogels are described as water-swollen polymers with specific 3-dimensional structures. The wet environment of hydrogels is useful for the regulation of inflammation, protection against infections, promotion of tissue regeneration, and the removal of wound exudates. For these reasons, they have been used to improve biomedical applications [11, 13]. There have been many research studies for making tissue-derived hydrogels used for treatment as grafts or as scaffolds for the same tissue. The compositions of the extracellular matrix of these tissue-derived hydrogels are similar to those of the origin or native tissue [11, 14]. The decellularized materials used for the preparation of hydrogels have a similar component to the tissue of origin, which improved the development of tissue-specific scaffolds for suitable cell-matrix interactions [15]. The decellularization and sterilization process can be performed by using physical or chemical or enzymatic protocols or by using a combination of these protocols [16, 17].

## 2. Materials and Methods

Thirty-six local breed healthy rabbits were used in this study to evaluate the effectiveness of urinary bladder submucosa scaffold and tendon-derived hydrogel scaffold on wound healing. They were subdivided into three equal groups; control group A, treated group with submucosal scaffold B, and the treated group with hydrogel scaffold group C.

The animal experiments were performed based on the Institutional Ethics Committee (IEC) approval and guidelines of the College of Veterinary Medicine/Fallujah University No. 22 at 14/11/2023.

### 2.1. Preparation of Decellularized Urinary Bladder Matrix

All urinary bladders were obtained from the slaughter houses. Fresh urinary bladders were collected as whole from slaughtered cows at the local abattoir, and the urinary bladder matrix (UBM) was prepared as a decellularized scaffold. The decellularization process of the urinary bladder was carried out according to Eberli et al. [18] and Alkhilani et al. [19].

### 2.2. Preparation of Tendon-Derived Hydrogel (TDH)

The decellularization process of the tendon was carried out according to [11, 20]. The tendon was harvested from slaughtered sheep at local abattoir. The muscle tissues, epitenon, and the synovial sheath were accurately debrided. Then, the tendons were decellularized with 0.1% ethylene diamine tetraacetic acid (EDTA) for about 4 hours, then followed by 0.1% concentration of sodium dodecyl sulfate dissolve (SDS) in 0.1% (EDTA) for 24 hours with constant agitation at room temperature, then washed by phosphate buffered saline (PBS), and stored at −80°C. The frozen decellularized material was thawed at room temperature and lyophilized and then milled into powder by Wily mini mill. The prepared powder was stored at 4°C until needed for the preparation of the gel, and the preparation of the hydrogel had been done according to [11, 21]; the prepared powder was enzymatically digested by the addition of 1 mg/ml of pepsin's solution with 0.02M hydrochloric acid (HCl) and sterile water, the concentration of the prepared material was 20 mg/ml, and its pH was 2.2. Then, it was digested for 24 hours with constant stirring at room temperature. The liquid that resulted was cooled on ice while raising the pH to 8 by using sodium hydroxide to deactivate the pepsin, and then the pH was lowered to 7.4. The concentration of the salt was adjusted by the use of 10X PBS to reach to the isotonic solution. The hydrogel was stored in a refrigerator at 4°C with a pH of 7.4.

### 2.3. Surgical Technique

**Figure 1:**
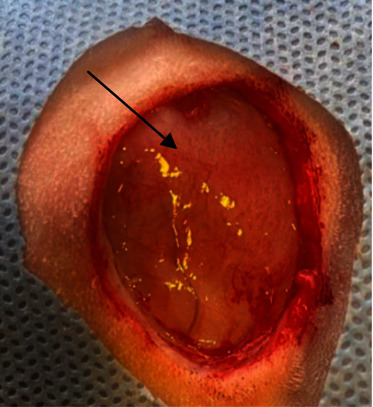
The induced surgical wound two-centimeter in diameter (black arrow).

**Figure 2:**
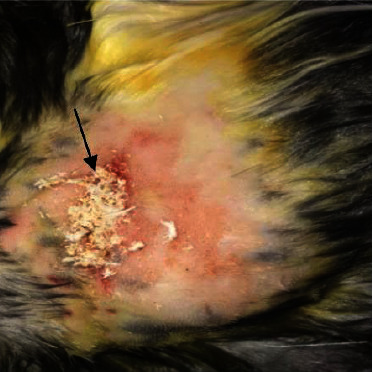
The application of urinary bladder submucosa scaffold to the induced wound in group B (black arrow).

Thirty-six adult rabbits (8–12 months), with an average weight of 2.0–2.5 kg, were used for the experiment, which were divided randomly into three equal groups (12 animals for each group). All groups were subjected to induced full thickness skin injury (2 cm) in diameter at the right lateral aspect of the abdominal wall under general anesthesia by using a combination of ketamine and xylazine (35 mg/kg B.W and 5 mg/kg B.W, I/M), respectively [22], all animals were monitored during and postoperatively until full recovery from anesthesia. The right lateral abdominal side was prepared aseptically for the creation of (2 cm) full thickness skin wounds as shown in (Figure 1). The experimental animals in the 1^st^ group (A) were sutured without the application of any substance as a control group, the rabbits of the 2^nd^ group (B) were sutured and treated with the application of decellularized urinary bladder submucosa scaffolds as shown in (Figure 2), and in the 3^rd^ group (C), they were sutured and treated with the application of tendon-derived hydrogel. The surgical incision of the animals was sutured with (Silk No. 2/0) and simple interrupted suture technique, with changing of the bandage each another day during the time of the experiment. Clinical, morphometrical, and histopathological evaluations have been done to assess the healing of the wounds in all groups during the time of the experiment. Daily clinical examination of animals has been performed during the time of the study, and the percentage of wound contraction was calculated during the period of the study. The histopathological examination was performed on the 8th, 14^th^, 21st, and 24^th^ day postcreation of wounds for all groups.

Biopsies were obtained from the sites of the wound bed and periphery, using the excisional scalpel blade technique. Samples were fixed in 10% neutral formalin solution for 72 hours after irrigation with normal saline, after which they were dried in graded levels of ethyl alcohol (70%, 80%, 90%, and 100%), xylene, and melting paraffin using an automatic processor. Then, they were embedded in the paraffin block and sectioned by a microtome at 5–7 micrometers thick. The slides were stained with a hematoxylin-eosin (H&E) dye according to [23].

## 3. Results

### 3.1. Macroscopic Observation

**Figure 3:**
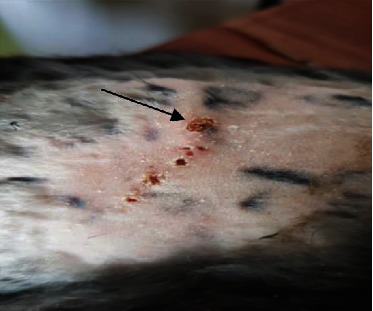
Macroscopic appearance at the 21^st^ postwounding day in group B shows clearly improved wound healing (black arrow).

**Figure 4:**
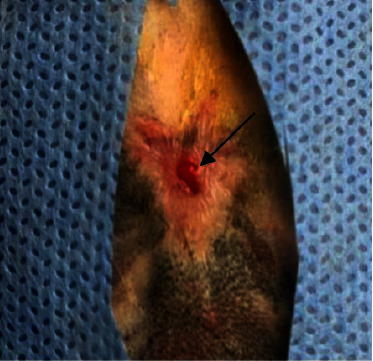
Macroscopic appearance at the 21^st^ postwounding day in group C shows the healing activity and the reduction in the wound's size (black arrow).

In the treated group with urinary bladder submucosa, clearly improved wound healing activity has been observed when compared to the control group and tendon-derived hydrogel group of animals. It also showed significant reduction in the wound area as in Figures 3 and 4.

### 3.2. Microscopic Appearance

#### 3.2.1. Control Group (A)

**Figure 5:**
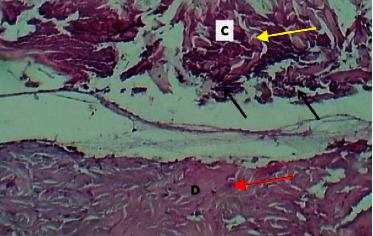
Micrograph at the 8^th^ day after wounding in group A shows the presence of inflammatory cells (black arrow), fibrin deposit (red arrow) with the presence of edema (yellow arrow), and clear separation between the epidermis and the dermis (H&E.100x).

On the eighth postwounding (P. W.) day, the histopathological features at the site of wound section were represented by formation of marked epidermal damage which was replaced by thick fibrin clot, necrotic tissue, which was separated from the dermis by inflammatory cells, and the dermis showed intact collagen bundles (Figure 5).

**Figure 6:**
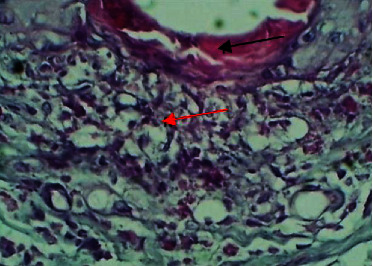
Micrograph at the 14^th^ day after wounding in group A shows the presence of remnant of fibrin clot (black arrow) and dermis degeneration and presence of necrosis (red arrow) (H&E.400x).

At the fourteenth P. W. day, the histopathological features at the site of wound section were showed remnants of the fibrin clot, dermal edema, presence of necrotic area in the collagen bundles, and penetration of leukocytes and bacterial colonies (Figure 6).

**Figure 7:**
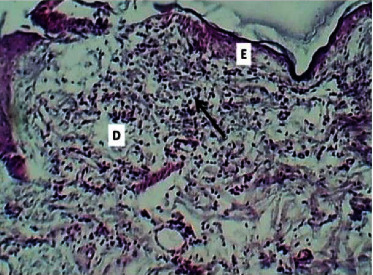
Micrograph at the 21st day after wounding in group A shows the presence of poor epithelization (E), moderate dermatitis (black arrow), and presence of edema (D) (H&E.100x).

**Figure 8:**
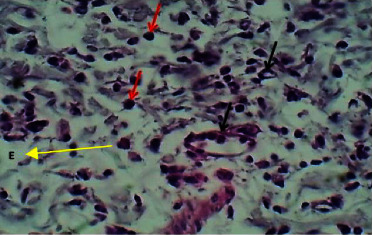
Micrograph at the 21^st^ day after wounding in group A shows the presence of dermatitis with mild dermal edema (yellow arrow) and infiltration of lymphocytes and macrophages (red arrows) within necrotic collagen bundles, and there was mild angiogenesis (black arrow) (H&E.400x).

At twenty first P. W. day, the histopathological features at the site of wound section were represented by poor epithelization. The dermis showed moderate dermatitis which was characterized by dermal edema, infiltration of mononuclear leukocytes mainly lymphocytes, and macrophages within necrotic collagen bundles, and there was mild angiogenesis (Figures 7 and 8).

**Figure 9:**
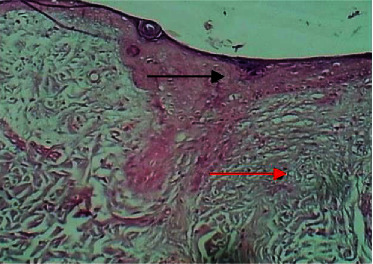
Micrograph at the 24^th^ day after wounding in group A shows the presence of complete epithelization (black arrow) and dermal fibroplasias (red arrow) (H&E.100x).

At twenty-fourth P.W. day, the histopathological features at the site of wound section were represented by complete epithelization. The dermis revealed marked fibroplasia without formation of hair follicles or sebaceous glands, and there were no inflammatory infiltrates (Figure 9).

#### 3.2.2. Group of Urinary Bladder Submucosa Scaffold (B)

**Figure 10:**
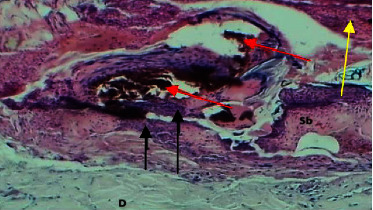
Micrograph at the 8^th^ day after wounding in group B shows the presence of fibrin deposit (yellow arrow), hemorrhage (red arrows), and necrotic material and inflammatory cells (black arrows) (H&E.40x).

**Figure 11:**
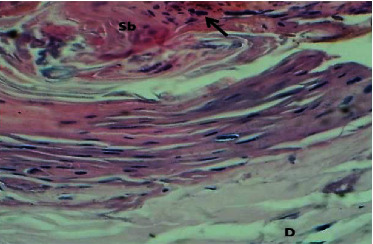
Micrograph at the 8^th^ day after wounding in group B shows the presence of necrotic collagen fibers with few inflammatory cells (black arrow) (H&E.400x).

At the eighth P. W. day, the sections of the skin showed marked epidermal damage which was replaced by fibrin deposits and dressing submucosal tissue, necrosis of collagen bundles, and little hemorrhage. The part of the muscularis was attached to the dermal tissue which revealed normal appearance without changes (Figures 10 and 11).

**Figure 12:**
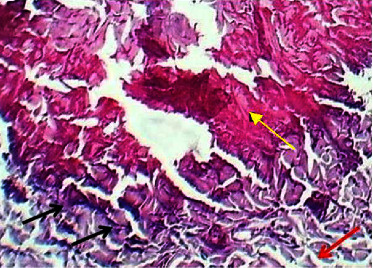
Micrograph at the 14^th^ day after wounding in group B shows the presence of degeneration and necrotic tissue (yellow arrow), line of reepithelization (black arrow), and mild degeneration of dermal collagen bundles (red arrow) (H&E.100x).

**Figure 13:**
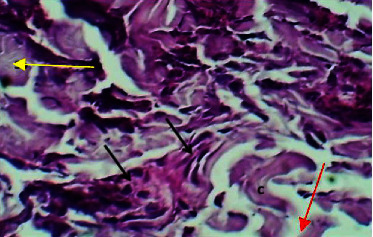
Micrograph at the 14^th^ day after wounding in group B shows the presence of degeneration and necrotic tissue (yellow arrow), line of reepithelization (black arrow), and mild degeneration of dermal collagen bundles (red arrow) (H&E.400x).

At the fourteenth P. W. day, sections of the skin showed massive degeneration and necrosis of dressing submucosal tissue and there was a line of reepithelization separating the dermal tissue. The dermis showed mild degeneration of collagen bundles. The section revealed mild infiltration of inflammatory cells (Figures 12 and 13).

**Figure 14:**
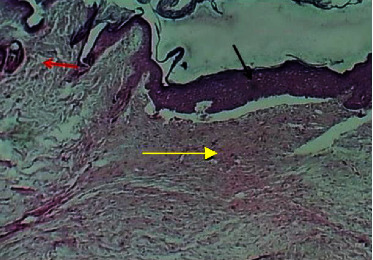
Micrograph at the 21^st^ day after wounding in group B shows the presence of epithelization (red arrow) and immature collagen fibers and angiogenesis (yellow arrows) (H&E.100x).

**Figure 15:**
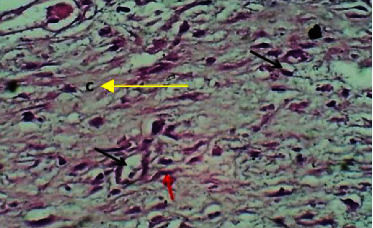
Micrograph at the 21^st^ day after wounding in group B shows the presence of new production of immature collagen fibers (yellow arrow), newly formed capillaries and angiogenesis (black arrow), and presence of fibroblasts (red arrow) (H&E.400x).

At the twenty first P. W. day, sections of the skin showed well epithelization which was characterized by the marked thick epidermis. The dermis showed active fibroplasia associated with newly production of immature collagen fibers and angiogenesis. The section also revealed few lymphocytes and no hair follicles neither sebaceous gland (Figures 14 and 15).

**Figure 16:**
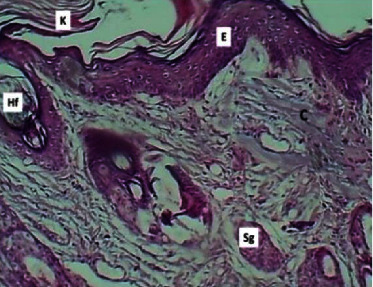
Micrograph at the 24^th^ day after wounding in group B shows the presence of thick keratinized stratified squamous epithelium (K), epidermis (E), mature collagen bundles (C), hair follicles (Hf), and sebaceous glands (Sg) (H&E.100x).

At the twenty-fourth P. W. day, sections of the epidermis showed thick keratinized stratified squamous epithelium. The dermis showed active fibroplasia associated with continuation production of collagen fibers and angiogenesis. The section also revealed no inflammatory infiltrates and marked hair folliculogenesis with the formation of sebaceous glands (Figure 16).

#### 3.2.3. Group of Tendon-Derived Hydrogels

**Figure 17:**
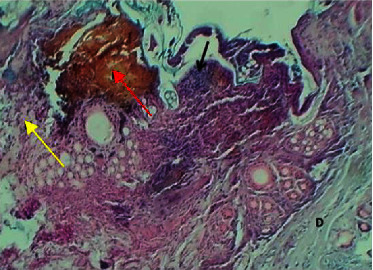
Micrograph at the 8^th^ day after wounding in group C shows the presence of necrotic tissue (yellow arrow), severe hemorrhage (red arrow), and presence of inflammatory cells (black arrow) (H&E.100x).

At the eight P. W. day, sections of the skin showed severe epidermal damage that was replaced by a very thick layer of necrotic tissue associated with severe hemorrhage and infiltration of inflammatory cells, while the dermis revealed normal appearance (Figure 17).

**Figure 18:**
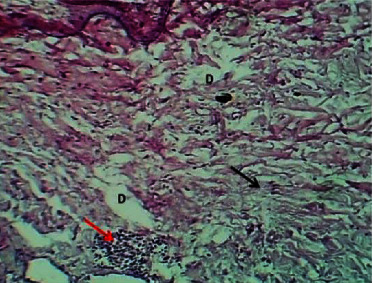
Micrograph at the 14^th^ day after wounding in group C shows the presence of necrosis of collagen bundles (black arrow), depletion of dermal tissue (D), and presence of inflammatory cells (red arrow) (H&E.100x).

At the fourteenth P. W. day, the sections of the skin showed no signs of reepithelization and degeneration and necrosis of the collagenous tissue have invaded the dermal tissue. The dermis showed mild dermal depletion of collagen with infiltration of inflammatory cells (Figure 18).

**Figure 19:**
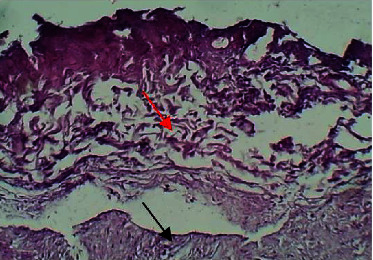
Micrograph at the 21^st^ day after wounding in group C shows the presence of necrosis of collagen bundles (black arrow) and depletion of dermal tissue (red arrow) (H&E.100x).

At the twenty-first P.W. day, the section of the skin showed necrotic collagen bundles with depletion as shown in (Figure 19).

**Figure 20:**
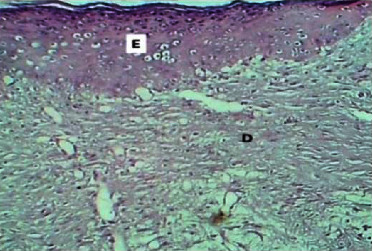
Micrograph at the 24^th^ day after wounding in group C shows the presence of epithelization (E), and the dermis (D) showed active fibroplasia and marked hair follicles with the formation of sebaceous glands (H&E.100x).

At the twenty-fourth P.W. day, the sections of the skin showed complete epithelization. The dermis revealed marked fibroplasia with the formation of hair follicles or sebaceous glands, and there was no inflammatory infiltrate (Figure 20).

## 4. Discussion

In the present study, efforts have been made for providing a good technique in the wound healing process depending on gross and histopathological evaluation of healing capability by the use of urinary bladder submucosa and hydrogel fabricated from tendon on the skin healing process in rabbits.

### 4.1. Macroscopically

The gross macroscopically examination showed that all animals tolerated the surgical procedures well and survived healthily during the study period. This result is consistent with that of the other researchers who served the rabbits as a good model for the skin wound healing study. All animals well tolerated the surgical procedures, and the wounds were healed without any complications. This was achieved from the aseptic technique, postoperative follow-up, and good management of the animals. No evidence of rejection at the injured area was seen, which explains that the decellularized materials are durable, biocompatible, and immunologically inert. These observations were close to the authors in [16, 24] who verified similar signs in an injectable tendon-derived hydrogel in the Achilles tendon of Wistar rats and with the authors in [11] who verified that the use of tendon-derived hydrogel in the treatment of tendon defect in rams are durable, biocompatible, and immunologically inert. Also, these results agreed with the authors in [19] who indicated that the decellularization methods are beneficial in sterilization of the prepared urinary bladder submucosa used in the treatment of tendon defect in rabbits.

### 4.2. Microscopically

In general, the microscopic examination of skin sections from three distinct treatment groups revealed varying patterns in the postwounding days. These observations provide detailed insights into the microscopic changes during the wound healing process in each group over time.

At the eighth postwounding day, the results showed the presence of inflammatory cell infiltration in all groups indicating an early response to injury, but they are severe in group C as compared to the other two groups based on the number of inflammatory cells and exhibited a distinct response to wound healing. Fibrin clot was intense in group C, but it was moderate in group B. In all groups, there were epidermal damage and collagen necrosis and different degrees of hemorrhage. Despite these initial challenges, the wound healing process progressed, achieving complete epithelization and marked fibroplasia by the twenty-fourth day. These results did not show significant difference in the healing process, although there is a difference among the severity of inflammatory cell infiltration, fibrin deposition, and epidermal damage. This may be due to the short period after wounding and the onset of the healing process in all groups. It is important to note that the presence of some differences indicates the effect of the treatment by substances used in groups B and C. These results are consistent with what was shown by [25] that the inflammatory cells during the stages of wound repair presented through the hemostasis and coagulation phase and characterized by the presence of neutrophils in the early stage and monocytes in the late stage and with [26] which found the presence of evidence that the severity of inflammation determines the time required for the scar formation, and there is evidence that the amount of inflammation determines the extent of scar formation.

Also Eming, 2007, said that the inflammatory response to wound is important for providing the growth factor and cytokine signals that are accountable for cell and tissue movements, which are critical for the following repair process in mammalians [27].

At the 14^th^ P. wounding day, the fibrin clot still appeared in group A, with dermal edema and infiltration of inflammatory cells with no signs of reepithelization, but the infiltration of inflammatory cells was mild in treatment groups in a line of reepithelization which was indicated good healing compared to group A, and this is consistent with [28, 29], and they mentioned that the development of the capillary will activate the migration of the local fibroblasts through the network of fibrin, angiogenesis, neovascularization, and commencement of reepithelization of the edges of the wound. Moreover, Eckes and his group [30] mentioned that fibroblasts are the dominating cells in this phase, which activate collagen production and production of extracellular matrix substances.

Acellular matrix contains the dermal elements with the collagen matrix and fibroblast on it, which work for the stimulation and secretion of the growth factor by which the epithelialization of the wound is achieved. Acellular matrix releases cytokines, and growth factors assimilate in dressings as bioengineering has the ability of modifying them to their environment [31].

At the 21^st^ postwounding day, the healing picture was clear in treatment groups through the appearance of good epithelization and active fibroplasia associated with the new production of immature collagen fibers and angiogenesis, compared with the control group which still shows inflammatory cells with the presence of poor epithelization and mild angiogenesis. The emergence of advanced healing in these groups is due to the presence of ECM scaffold, which provides the basic parts for restoration and healing. This is consistent with the authors in [32], and they demonstrated that ECM is a perfect biomaterial used for the reconstruction of the tissues that were used in surgery as a mesh and scaffold to bridge the edges of the injured tissues. The basic components of tissue's extracellular matrix are collagen, laminin, and soluble growth factors. These biomaterials supply the structural supports which adjust the attachment and behavior of the cells during the processes of tissue repair [33]. Extracellular matrix is an inductive scaffold for functional tissue reconstruction [32]. Parmaksiz, 2015, showed that ECM biomaterials reserve collagen fibers, glycosaminoglycans, and many of growth factors such as basic fibroblast growth factor (b-FGF), transforming growth factor-*β* (TGF-*β*), and vascular endothelial growth factor (VEGF) which play an important role in rebuilding by chip in the cell behavior [34].

At the 24th postwounding day, the histopathological study showed advanced stages of healing through the occurrence of thick keratinized stratified squamous epithelium, active fibroplasia associated with continuation production of collagen fibers, angiogenesis and the absence of the appearance of inflammatory cells, and marked hair folliculogenesis with formation of sebaceous glands in treatment groups B and C. The absence of inflammatory cells and the appearance of hair follicles and sebaceous glands in the treated groups indicate that the healing process has reached the remodeling stage. The results of the control group showed that there are also advanced stages of healing, but they did not reach the degree of healing in the two treated groups B and C. The formation of the ECM represents another important step as it provides a scaffold for cell adhesion and critically regulates and organizes the growth, movement, and differentiation of the cells within it [30, 35]. The fibroblast is, therefore, the precursor of the provisional wound matrix on and in which the respective cell migration and organization takes place [36].

Remodeling is the last phase of wound healing and occurs from day 21^st^ to up to 1 year after injury. The formation of granulation tissue stops through apoptosis of the cells. A mature wound is, therefore, characterized as avascular as well as acellular [35, 37]. Moreover, different changes are presented during the maturation phase of wound healing, such as the replacement of collagen type III by collagen I, and oriented in small parallel bundles. Eckes and Barker found that the ECM regulates the growth and differentiation of cells and provides a scaffold for the adhesion of the cell [30, 35].

While there were observable differences in the microscopic appearance among the groups, the overall healing process did not significantly differ. All groups eventually achieved complete wound closure, albeit with variations in the timing and intensity of inflammatory responses, fibrin deposition, and fibroplasia. The presence of hair follicles and sebaceous glands in groups B and C suggests a more mature and organized tissue regeneration compared to the control group.

## 5. Conclusions

In conclusion, the microscopic analysis of wound healing in different treatment groups reveals distinctive patterns in the early and late phases of tissue repair. The use of urinary bladder submucosa scaffold and tendon-derived hydrogels appears to enhance wound healing.

Further studies with extended observation periods and molecular analyses are needed to elucidate the underlying mechanisms and validate the clinical relevance of these findings.

## Data Availability

The preparation of hydrogels and results' data used to support the findings of this study are included within the article and have been deposited in the Biochemical and Cellular Archives, 2020, 20(1), pp. 1591–1600.
